# Improving Mental Performance in an Athletic Population with the Use of Ārepa^®^, a Blackcurrant Based Nootropic Drink: A Randomized Control Trial

**DOI:** 10.3390/antiox9040316

**Published:** 2020-04-15

**Authors:** Natalie Gibson, Dane Baker, Alice Sharples, Andrea Braakhuis

**Affiliations:** 1 Discipline of Nutrition & Dietetics, Faculty of Medical and Health Science, The University of Auckland, Auckland 1010, New Zealand; ngib846@aucklanduni.ac.nz; 2 High Performance Sport, AUT Millenium, Auckland 0632, New Zealand; dane.baker@hpsnz.org.nz; 3 Vodafone Warriors Rugby League, Mt Smart Stadium, 2 Beasley Avenue, Auckland 1061, New Zealand; alice.sharples@gmail.com

**Keywords:** cognition, polyphenols, blackcurrant, sport

## Abstract

A range of dietary bioactive ingredients have claimed to improve mental clarity and reduce fatigue, including blackcurrant, pine bark, and l-theanine. These active ingredients provide a good source of dietary polyphenols which could be useful in reducing mental fatigue in a sports setting. The aim of the investigation was to test the effect of Ārepa^®^ a blackcurrant-based nootropic-drink also containing pine-bark and l-theanine (BC+), on mental clarity in a sport setting. Twenty-three rugby league players completed a cross-over design, randomized, double-blind, controlled trial. Intervention and control phases lasted 7 days, with a washout in between. Cognition was assessed pre and post intervention following a standardized training session. Our study found the total score, accuracy, and average time per response scores improved significantly more after drinking the BC+ drink (*p* = 0.001, 0.003, and 0.043 respectively). The BC+ improved the perception that participants were reliable (*p* = 0.02) and less distracted (*p* = 0.03), while placebo supplementation increased participant perception they could control their nervousness (*p* = 0.03). Thematic analysis of post-trial questionnaire indicated participants found the BC+ sour, most reported no side effects, and opinion on which drink was more effective was not unanimous. The results indicate that the BC+ drink may be useful for athletes.

## 1. Introduction

The modern-day athlete is interested in stimulating changes in performance and improving training accuracy and effectiveness thereby increasing the chance of winning [1]. Athletes place a large emphasis on improving physical ability as a method to improve performance. While it is recognized physical capability is a major factor in optimal sporting performance, sporting professionals have become more aware of the role the mind plays in influencing cognitive performance. This holds particularly true for sports, such as rugby league, that involve decision making, coordination, and the use of a variety of skills. Optimal cognitive function is beneficial for athletes, not only because performance in many sports requires motor control, coordination, decision making, timing and teamwork, but further, is important in reducing the stress involved with athletic competition. As an indirect effector of cognition, sport-related stress stems from a number of sources including the physical stress of the movements needed to participate in the sport, the stress of competing or the pressure exerted from coaches and team members [2]. The importance of elevated stress in athletes, is the adverse physiological changes that occur in response to increased stress hormones. Stress hormones, such as cortisol, impair immune function, inhibit anabolic pathways, and promote catabolism, driving an increased risk of injury/illness, impaired ability to adapt and repair muscles, as well as increased perception of pain and fatigue [3]. Interventions that may independently reduce excess stress may indirectly assist in improving cognitive outcomes in high pressure sporting situations.

With emerging evidence suggesting anthocyanin-rich blackcurrants may have a role in preventing cognitive decline [4] and enhancing physical performance [5,6], there is interest that the richly colored berries may elicit further benefits such as acute improvements in cognition. Alongside the increasing evidence for blackcurrant polyphenols, pine bark extract has also shown to improve the speed of response for the spatial working memory and immediate recognition tasks in humans [7,8], although most research has tested non-athletic, older adult participants. Nootropics are defined as substances that may improve cognitive function, particularly executive functions, memory, creativity, or motivation, in healthy individuals. For many years caffeine has been used as a successful nootropic aide, as moderate doses of caffeine can improve cognitive function, including vigilance, learning, memory, and mood state [9]. However, caffeine can become addictive, hence the search for alternative nootropic agents.

A recent narrative review highlighted the physiological mechanisms behind the potential of blackcurrant polyphenols to influence cognition [10] stating acute intake alters vasodilation and increases peripheral and cerebral blood flow. Recent evidence from controlled human trials demonstrate the link between polyphenolic flavonoids and improvements in cognitive function in healthy individuals [11] with effects ranging from improvements in mood and working memory [12] to improvements in simple reaction time [13]. Blackcurrants are a rich source of polyphenolic flavonoids, in particular, anthocyanins [10]. With limited research available on cognitive effects of blackcurrant in non-impaired adults, and no evidence in a specific athletic population, this study aimed to investigate the effect of a pre-existing blackcurrant-based supplement Ārepa^®^ (BC+) advertised as a “nootropic brain drink” on mental performance in a sporting setting.

## 2. Materials and Methods 

### 2.1. Subjects

Thirty male and 20 female rugby league players were invited through their rugby league club to participate in the study. The subjects typically trained for 90 min twice a week and play one 80 min rugby game each week. The trainings consisted of field-based drills and skills practice, and occasionally included additional time discussing strategic aspects of upcoming games. The trial took a total of 3 weeks conducted pre-season in a stable stage of the training calendar. The timing of the trial was specifically chosen as expected training loads were the same during intervention and control time periods, in consultation with coaching staff. Prior to the study, all prospective subjects attended a post-training presentation to inform each player of the testing procedures and possible risks involved in the study, following which each participant gave written consent. The cognitive test was demonstrated and trialed by participants. Ethical approval was granted from The University of Auckland Human Participants Ethics Committee (reference no 022387). To be eligible to participate in the trial, each player needed to be available to complete Stroop testing on each of the four designated post-training test dates, and be free of known cognitive impairment.

### 2.2. Experimental Design

The study used a placebo-controlled double-blinded randomized crossover design to investigate the effect of blackcurrant (BC+) drink on mental clarity in a sporting setting. Participants were individually randomized using a computer-generated sequence to a 7-day period of either BC+ supplementation (1:1), or an appearance and taste matched inactive control beverage (PL). The randomization and product labelling occurred separately to the data collection to ensure the research personnel in contact with the participants were blinded to the intervention. Following the first 7-day period of supplementation (phase one), each player received no supplemental beverages over a 10-day wash-out phase. Afterwards, each player received the alternate beverage for a second 7-day period (phase two). This design ensured each player received both PL and BC+ supplementation for 7 days, and could therefore act as their own control. Randomization and labelling of supplementation was conducted by a research assistant uninvolved with data collection and player contact to ensure this trial remained double-blinded.

### 2.3. Sample Size

The estimated sample size of 20 participants was calculated which would enable the detection of a smallest worthwhile treatment effect. In the sample size calculation, the between-subject standard deviation was determined to be 6.0 (neurocognitive testing score) and the re-test reliability, 0.87 [13] and these values calculated a typical error of 0.36. A greater number of players were recruited to allow for dropouts.

### 2.4. Primary Measure

The primary measure of mental clarity in this study was a Stroop test. Briefly, it consists of a set of stimuli with words displayed in a single color and that form the name of another color, for instance: the word “blue” printed in green color. When the participant is asked to answer on the color of the word and ignore its identity (the word–color interference effect), the automatic processing of the word identity is inhibited due to less automated processes, such as the color used to print the word [14]. Computerized versions are considered more accurate than paper-pen versions [15]. The Stroop test appears to be valid in assessing food–nutrient interactions in assessing aspects of cognition in clinical trials [16]. Previous research reports a link between Stroop test results and success in ball sport activities [17]. The test was administered to players using an iPad tablet, on-site following the evening training session. The importance of this time window was that it captured the participants in a fatigued state, when mental clarity would be its most vulnerable, and was approximately 90 min after consumption of their assigned test beverage. A 60 s Stroop test duration was chosen on the basis it would capture a cognitive response, however, be short enough to keep the participant burden low. Each participant was tested following their 90 min training session in the familiarization, the evening before beginning supplementation (baseline), and following a complete week of supplementation (final). This was repeated for both phases one and two of the study, so that each player would have a baseline and follow-up measure for both control and BC+.

Testing was conducted in the clubhouse training rooms, on each occasion, in the evening. The rooms contain tables and chairs and participants were asked to be seated 2 m from a wall during testing. There was standard indoor lighting, and the iPad was set to standard visual display. Research staff and participants were the only people present, and there was no external noise. Test results were captured and saved by taking a screenshot of the results that were displayed once each participant had completed the test. Each screenshot was identified by recording which tablet the player used to complete the test (either A, B, C) and the order in which participants used each tablet, generating an individual code for each participant each session (e.g., B5). Each test generated a total score, total responses, accuracy percentage, average time per score average time per response, and score within each quarter of the test.

### 2.5. Secondary Measure

A validated mental toughness assessment (MTQ48) was used to gather further information on each player’s subjective mental status throughout each phase. At two intervals during each 1-week supplementation period, a blinded researcher sent the modified questionnaire via TXT message to each subject. A follow up reminder text was sent to all non-responders to increase the response rate. A third MTQ was given to the players on paper to complete before training which began on the final day of each phase. The MTQ48 was modified to include 11 of the most relevant questions rather than the full 48 questions and to reduce participant burden. Both the MTQ48 and subsections of the questionnaire have been validated elsewhere [18,19]. Participant answered the questionnaire creating a score from 1 to 5. Questions a, b, c, f, g, h, i, j, k measured a positive mental attribute, where answer 5 was the most desirable outcome. Questions d and e measured negative outcomes, where 1 was the most desirable outcome. Therefore, an inverse score was created for questions d and e to make data comparable for analysis. In each phase of the trial, participants were expected to submit a total of three responses, where participants had one response missing, data was still included in analysis. If a participant had failed to report two responses, this left only one response remaining and did not allow for a comparison to be made, data was therefore excluded. Participant responses were screened before inclusion in analysis to detect answers that contained an incorrect number of responses or where answers had not been reported truthfully. This included answers where participants reported the answer to all questions a–k was 5, which was considered to be false given that d and e were inversely weighted.

### 2.6. Supplement Delivery

One researcher (AB) collected the intervention and control product from the distribution center and labelled the drink with the participant name, using the pre-prepared randomization. Following the product labelling, the researcher (AB) did not have contact with the participants. Named supplements were distributed to players by a blinded research assistant (NG) on site of their training location. Following completion of a baseline Stroop test, each participant was supplied with six unlabeled, identical supplement vessels containing either BC+ or a PL beverage. Participants received TXT message communication daily, at approx. 6pm, from a blinded research assistant to remind them to consume their daily supplemental beverage. Participants were encouraged to send confirmation they had consumed their beverage, to allow for detailed player tracking and records. On the seventh day of each phase, a blinded research participant handed each player their final supplement before they began training. This was to ensure each player consumed their beverage at the same time, prior to the Stroop that was conducted following training.

### 2.7. Dietary Data

Each player was asked to complete a 1-day diet record during each phase of the trial (twice total). To reduce participant burden and increase the accuracy of dietary data supplied, players were instructed to take photos of each meal, snack, or beverage they consumed within that given day. Photos were sent to a blinded research staff using free messaging platforms self-selected by the participant, including Viber, WhatsApp, and Facebook Messenger. In circumstances where this was not possible or photos were missing, a researcher sent TXT message responses requesting detailed reports of the missing food and beverage items.

### 2.8. Subjective End of Trial Questionnaire

One week following the completion of phase two of the trial, each participant was contacted via TXT to complete a questionnaire on their personal perception of the effectiveness of the supplemental beverage. Participants were encouraged to identify any side effects, how effective they perceived the drinks to be, and in which phase they felt a greater effectiveness.

### 2.9. Intervention and Control Products

The supplement used was a pre-existing product that was marketed as enhancing cognition, commercially branded as Ārepa™. The BC+ drink contained the following ingredients blackcurrant juice, apple juice, water, flavors, blackcurrant extract, decaffeinated green tea extract, citric, l-theanine, pine bark extract (Enzogenol). The BC+ and PL drinks were iso-caloric, containing 52 kJ, 0.1 g protein, 0.1 g fat, 7.7 g carbohydrate per 100 mL of drink. The intervention product also contained total polyphenols (465 mg) and anthocyanins (155 mg), l-theanine (80 mg), pine bark extract (50 mg), and vitamin C (30 mg) per 100 mL. The polyphenol and anthocyanin content were independently assessed by the Cawthron Institute. Both intervention and control products contain no caffeine. To ensure the active ingredients did not deteriorate throughout the trial, product was produced in the days leading up to the trial, and stored in an industrial refrigerator until presented to the participant. Both drinks were presented to participants in identical dark purple tinted plastic bottles.

### 2.10. Analysis

Stroop test data was analyzed using GraphPad Prism software (version 7.03, San Diego, CA, US). Prior to analysis, the distribution of the Stroop test data was assessed using the D’Agostino and Pearson normality test. The Stroop test data pre-post within group comparison was analyzed using the relevant paired data test. In the event of non-normal data, normalization was attempted and *t*-test or the Wilcoxon test was conducted. The 3 days of MTQ data collection for each arm were analyzed using a repeated measures ANOVA for the intervention. Statistical significance was set at 0.05. To ensure all data were appropriate to analyze, the ROUT coefficient Q was calculated to determine data outliers (Q = 1%).

#### 2.10.1. Dietary Data

Dietary data was received as a combination of photographic and written records. The data was analyzed by a student dietitian to assess if the record was likely to be a complete record on the basis it has to contain a minimum of approximately 1600 calories. The eligible diet records were analyzed to determine the number of serves of grain, fruit, vegetable, dairy, and protein foods each participant consumed. When used as the primary record of dietary intake, images can provide valid estimates of energy intake [17,20], but may underestimate other nutrients. The food diaries were skimmed for food items high in anthocyanin content, analyzed using the Phenol-Explorer database. The results are not presented in the table as anthocyanin intake is likely underestimated.

#### 2.10.2. Subjective End of Trial Questionnaire

A qualitative thematic analysis approach was taken to analyze the post-trial questionnaire. A general induction method [21] was used to analyze responses given by participants in response to the end of trial questionnaire. The responses were studied to identify meaningful segments and key themes. Themes emerged by grouping together common and recurrent ideas within the text. The text was re-read to group together and further refine themes and subthemes. Data was coded into themes and the frequency each theme appeared, along with the key quotes supporting each theme was recorded. A single researcher undertook qualitative analysis. A second researcher reviewed the methodology and confirmed identified themes.

## 3. Results

### 3.1. Participants

Of the 50 players who were invited to participate in the study, 23 rugby league players completed all aspects of the trial, and baseline characteristics of these participants can be found in Table 1. The team selected for participation were ranked as a top-tier team in the Auckland club rugby league series and would be considered sub-elite. A select number of players consented to participation, however, could not be included in the data analysis due to absence from one or more of the four designated post-training Stroop test dates, non-compliance with intervention, disassociation with the rugby team, or personal choice to withdraw. In the first phase of the trial 10 participants received PL and 13 received BC supplementation. Training load was matched during both phases of the trial to ensure it did not bias the results. The trial was conducted in pre-season before regular games commenced. Skinfold testing was conducted by the team nutritionist (AS) as routine practice and have been provided as descriptive information on the participants.

### 3.2. Stroop Test

A total of 23 participants completed the Stroop testing. On average, the total score increased by 4.7 following supplementation with BC+ compared to an increase of 3.6 following PL. Following supplementation with BC+, mean accuracy increased by ≈4%. This improvement in accuracy score was 3.1 more than the improvement in accuracy experienced following PL. The improvement induced following supplementation with BC was statistically significant across total score, accuracy, and average time per response (refer to Table 2).

### 3.3. MTQ Questionnaire

Twelve participants submitted at least two valid MTQ responses from each phase of the trial, rendering their results eligible for analysis. No significant difference was found between the mean 3-day total MTQ score of players that received either PL or BC+ supplementation (*p* = 0.40). When each individual question of the MTQ questionnaire was analyzed in isolation, it appeared BC+ supplementation improved participants perception that participants were reliable and less distracted, while PL supplementation increased participant perception they could control their nervousness (refer to Table 3).

### 3.4. Dietary Data

While all participants were encouraged and reminded to provide a full diet record during both phases of the trial, only 14 participants provided records that appeared to be complete and suitable to be analyzed. In general, participant diets were low in vegetables, fruit and grains, and high protein foods (see Table 4).

All diets were screened for external anthocyanin intake. Two participants indicated consumption of ½ cup of frozen blueberries regularly, this is equivalent to 80 g of blueberries, which contain approximately 150 mg anthocyanins. The remaining diet records did not contain any anthocyanin containing foods, so it is assumed baseline anthocyanin intake in these individuals is too low to measure. As participants indicated they consumed equivalent amounts during both the BC+ and PL consumption period, it is assumed the baseline anthocyanin was controlled.

### 3.5. Subjective End of Trial Questionnaire

The majority of participants who responded to the prompt regarding side effects of consuming the drinks reported no side effect associated with supplementation (92% of participants who discussed side effects). A total of 76% of participants agreed the PL beverage tasted superior, commonly describing the drink as “sweeter” and/or “smoother” compared to the BC+ beverage, which was commonly identified as “sour” and/or “bitter”.

The questionnaire asked participants to comment on which drink either BC+ or PL they felt had contributed to a greater outcome. Participants remained blind to which week they had received PL and BC+, and were rather were asked to note the differences observed between each 1-week phase. Analysis indicated there was no unanimous opinion between participants, as six participants perceived the PL to be more effective, six noted both the PL and BC+ supplements amounted to similar outcomes, and just four participants indicated BC+ had been more effective (one participant did not comment). See Table 5.

## 4. Discussion

The outcomes from the current trial demonstrate the blackcurrant-based supplement Ārepa^®^ improved cognitive performance in rugby league players over a 1-week supplementation period. Statistically significant improvements to cognitive measures were observed when the Stroop change score of the BC+ group was compared to the change score following placebo. The improvement in Stroop accuracy in the participants was greater following 7 days of BC+ compared to placebo. On average the total score improved by roughly 10% after the BC+ phase compared to 7% following the placebo phase. While other studies have trended towards an improvement in cognition, this is the first to report positive findings in an athletic cohort.

Secondary to the cognitive outcomes, mental stress and toughness were monitored throughout the trial. A modified MTQ48 was included as part of this trial, to collect subjective measures of mental performance and of stress over the trial period [18,19]. The mean total score, calculated by combining each participant’s responses to all three questionnaires submitted over each 1-week period, was higher during BC+ than placebo, although the differences were considered insignificant. Of note, the BC+ improved the perception that participants were reliable (*p* = 0.02) and less distracted (*p* = 0.03), while placebo supplementation increased participant perception they could control their nervousness (*p* = 0.03).

Watson et al. [22] investigated similar blackcurrant supplements, comparing the effect of either cold-pressed “blackadder” blackcurrant juice, or anthocyanin-enriched “delcyan” extract, containing similar anthocyanin content in an acute dose. The authors concluded the blackcurrant supplement had a positive modulation of behavior when compared to placebo. The cognition outcomes included an improved rapid visual information processing accuracy, increased subjective alertness rating and attenuation of subjective mental fatigue following BC extract, and improved digit vigilance reaction time with blackcurrant juice at select time points. Stroop cognitive testing was included in this study, however, results did not amount to statistical significance.

A further study by Watson, Okello, et al. [23] investigated the role of blackcurrant only on cognition using similar cognitive performance testing to the current study. In particular, the CogTrack system was used to assess cognitive performance, coupled with collection of electroencephalography (ECG) data to investigate spectral power of slow wave delta (δ), theta (θ), alpha (α), and beta (β) brainwaves. This study utilized a small sample size of eight male participants and reported an increase in the reaction time when completing a choice reaction time task. The use of ECG data allowed for researchers to monitor the change in the different frequencies of brain waves and relate these to different levels of attention, fatigue, relaxation, or excitement. The results suggested participants had increased alertness and lower fatigue following consumption of blackcurrant compared to placebo, as indicated by reduction in increase in β power and reduction in α power. Furthermore, ECG data indicated the blackcurrant product may have reduced the stress or anxiety of the mental performance testing, as evidenced by the increased slow wave δ and θ powers and reduction in α power. The results suggest blackcurrant containing products have a nootropic effect beyond direct cognition, and may influence the stress/anxiety such that executive functioning is maintained under stress.

Previous research has eluded to a positive cognitive effect of blackcurrant products, however, until now have not been tested in athletes. Results from the current investigation suggest the product may prove efficacious in a sport setting, particularly to support optimal cognitive function after fatiguing exercise. One of the recurring comments in the post investigation questionnaire is that many athletes found the BC+ drink sour, suggesting powdered products might be a better option, or allowing athletes to become familiar with the taste of the drink prior to use in competition.

With regards to the dietary intake of the participants, the consistent trends across both groups provide insight into the dietary patterns of this population. The data indicate the diet of the participants were similar in both phases, however somewhat nutritionally inadequate, as evidenced by low intake of vegetables and grain foods. The effect of any nutritional supplement on a participant with a nutritionally inadequate diet is questionable, however perhaps more efficacious in this group. However, an alternative explanation is that participants were under-reported their dietary intake, a common problem when collecting food records [20,24].

The study has limitations, while the study was powered adequately to detect changes in cognition (Stroop), the study is likely to be underpowered to detect a change in mental toughness or diet given just 12 participants completed the MTQ questions and 14 completed the full dietary image data. The study also had a significant drop out rate from initial recruitment to final analysis. The primary reason for the drop out was participants leaving the club, moving to a different club, or simply failing to attend training sessions where testing occurred. The results presented may not be representative of the general athletic population as a result.

A positive outcome of the Subjective End of Trial Questionnaire was that the majority of participants agreed taking the BC+ had no reported side effects, with just one participant indicating minor side effects that may have been linked to a rapid ingestion of the beverage. This indicates the dosing used was effective at inducing a cognitive improvement, and importantly, tolerable for the participant population. Another key finding of this aspect of the trial, was the differing opinions between participants as to the drink they felt had been more effective. This could indicate the blinding procedures followed was effective, as participants could not distinguish between the placebo and BC+ beverages.

## 5. Conclusions

This small scale, short-term study resulted in a significant increase in cognitive function scores of rugby league players after 1 week of BC+ supplementation. These promising results suggests BC+ supplementation in a sporting setting may be beneficial. Furthermore, this study justifies the need for a longer-term, larger scale, and more sensitive investigation into the use of BC+ supplementation in a sports setting.

## Figures and Tables

**Table 1:** Baseline participant characteristics.

	Mean	SD
Age	28	5
Weight (kg)	97	21
Height (cm)	175	11
Weekly training (h)	4.5	4
Sum of eight skinfolds (mm)	132	48
% Male	83	n/a

Key: SD: standard deviation.

**Table 2:** Stroop test.

	PL (Mean (SD))		BC+ (Mean (SD))		Change Score Difference	
	Pre	Post	Pre	Post		
Total score	50.61 (11.8)	54.19 (9.1)	47.87 (10.9)	52.64 (8.8)	1.2	*p* = 0.001 *
Total response	52.09 (10.3)	54.65 (9.3)	51.61 (10.1)	53.96 (8.8)	−0.2	*p* = 0.559
Accuracy	96.49 (7.3)	97.09 (3.5)	94.12 (15.4)	97.83 (2.7)	3.1	*p* = 0.003 *
Time/score	1.264 (0.39)	1.138 (0.21)	1.35 (0.49)	1.173 (0.23)	−0.05	*p* = 0.063
Time/response	1.195 (0.24)	1.129 (0.20)	1.207 (0.25)	1.143 (0.21)	0.002	*p* = 0.043 *

PL: placebo drink; BC+: blackcurrant containing drink; * statistical significance.

**Table 3:** Mental toughness assessment (MTQ) questionnaire.

	PL (Mean (SD) 3-Day Total Score	BC+ (Mean (SD) 3-Day Total Score	Intervention Difference
Total Score	123.9 (15.6)	125.9 (15.4)	*p* = 0.40
A (Control)	11.4 (2.1)	11.9 (1.5)	*p* = 0.12
B (Ability)	11.1 (1.9)	12 (1.5)	*p* = 0.48
C (Reliable)	10.9 (1.6)	12 (1.4)	*p* = 0.02 *
D (Relaxation) ^a^	10.75 (2.5)	11.75 (2.3)	*p* = 0.03 *
E (Non-distracted) ^a^	10.5 (2.4)	10.7 (2.5)	*p* = 0.75
F (Coping with problems)	11.1 (1.4)	10.9 (2.0)	*p* = 0.73
G (Quick reaction)	11.25 (2.0)	10.92 (2.0)	*p* = 0.19
H (Clam under pressure)	11.3 (1.5)	11.5 (1.9)	*p* = 0.73
I (High mental effort)	11.0 (2.4)	11.1 (1.5)	*p* = 0.81
J (Control nerves)	12.2 (1.7)	11.3 (2.1)	*p* = 0.03 *
K (Adapt)	11.7 (2.3)	11.8 (1.7)	*p* = 0.64

BC+: blackcurrant drink; PL: placebo drink; * two-way ANOVA repeated measures, statistical significance; ^a^ inverse score.

**Table 4:** Dietary data.

	Recommendation	Phase One Av.	Phase Two Av.
Grain	6	5.3	4.1
Fruit	2+	1.5	1.9
Vegetable	3+	1.4	1.6
Dairy	2	1.5	1.4
Protein	1–2	4.3	4.5

**Table 5:** Thematic analysis of subjective results.

Themes	Total *n* = 17	Reference Quotes
PL more effective	*n* = 6	I felt better in the week 2 [PL] I felt ’better’ after the second drink [PL], I felt the second drink [PL] ’helped’ me more I felt the 1st drink [PL] help me more, mentally felt a lot more focus. Couldn’t really tell with the second batch [BC+] I felt more alert and better with week 1 [PL] than week 2 [BC+] I liked week 1 [PL] better I liked week 1’s drink [PL] I liked the batch I got in the 2nd phase [PL], I did feel like I wasn‘t switched on as much as the first week [BC+]
No difference between drinks	*n* = 6	I think it helped but there wasn’t a huge difference with my experience I felt similar benefits between week 1 and week 2 when taking the drinks Which one worked better? Not sure When I didn’t have the drink I felt a bit tired, lack of energy. Example when we had the week with no drinks I felt good after taking both drinks The drinks didn’t provide me anything different from one another was the same buzz each time! I preferred each drink on the same level
BC+ more effective and “worked”	*n* = 4	The first [BC+] I felt that the drink had a stronger effect on my concentration and focus. Second week [PL] not so much, felt like it was less effective in both focus and concentration. In the first week [BC+] I felt more switched on … I felt more energy but second week [PL] I felt normal… Definitely felt better in the first block [BC+], I felt sluggish in the second week [PL] I would take it again had multiple benefits. Week 1 worked [BC+], week 2 didn’t [PL] I think week 1 [BC+] worked a bit better for me in terms of the effects the drink had on me, compared to week 2 [PL] I felt better and definitely noticed a difference in week 1 [BC+]
PL tasted better	*n* = 13	I liked the taste in week 2 [PL] drink Preferred the taste of the 2nd [PL], 1st drink [BC+] almost too sour the taste was better in the second drink [PL] First batch of drinks [PL] tasted way better than second batch [BC+]. The 2nd batch [BC+] was more bitter The drink in week 1 [PL] was smoother than the other drink [BC+] it was quite bitter I like the sweeter [PL] not so sour drink best I liked the 1st [PL] week better it tasted better, 2nd week was a bit to sour and bitter for me… I wasn’t looking forward to taking the drinks on week 2 [BC+] mainly because of the taste I liked week 1’s drink [PL] Second batch was sour [BC+], first was more blended [PL] 1st week [BC+] they were very sour and left a burning feeling on my chest … The 2nd lot of drinks [PL] I got were very tasty and sweet and I wanted more… I enjoyed week 2 [PL]. I felt week 2 [PL] tasted smoother I liked the first week [PL] better, I felt it wasn’t as tangy… I liked week 1’s [PL] drink mainly because of the taste Definitely [preferred] the first week [PL] with the flavor drink. I didn’t like the second weeks one [BC+]
No side effects	*n* = 12 (*n* = 1 reported a side effect)	I didn’t have any side effects had no side effects that I noticed No side effects on either drink No side effects I don’t think there were any side effects No side effects No side effects Zero bad side affects I had no side effects no noticeable side effects I did not experience any side effects Not sure if there was any side effects.
Description of benefits experienced		Sharper more energy The energy and sharpness from it was great Reaction time was better confident and smart More focus More energy than usual… mental performance did increase and at times I felt more energetic and upbeat. More focused Mental performance definitely improved… good focus… sharper More alert Helped as being a teacher, things can be stressful Big change in my performance… Energy levels were peaking, focus on the tasks during trainings was clear, noticed it helped stabilize my mood… Felt confident in myself, ability wise and appearance wise Felt excited I felt I wasn’t so edgy which made me concentrate a lot better I had good training when I had the drink. Particularly in mind clarity and energy levels
